# Eating Behaviors and Overweight among Adolescents: A Population-Based Survey in Japan

**DOI:** 10.1155/2013/717942

**Published:** 2013-07-17

**Authors:** Hirotaka Ochiai, Takako Shirasawa, Tadahiro Ohtsu, Rimei Nishimura, Aya Morimoto, Hiromi Hoshino, Naoko Tajima, Akatsuki Kokaze

**Affiliations:** ^1^Department of Public Health, Showa University School of Medicine, 1-5-8 Hatanodai, Shinagawa-ku, Tokyo 142-8555, Japan; ^2^Division of Diabetes, Metabolism and Endocrinology, Department of Internal Medicine, Jikei University School of Medicine, 3-25-8 Nishi-Shinbashi Minato-ku, Tokyo 105-8461, Japan; ^3^Jikei University School of Medicine, 3-25-8 Nishi-Shinbashi Minato-ku, Tokyo 105-8461, Japan

## Abstract

*Objectives*. The aim of the present study was to investigate the relationship between eating behaviors and overweight among population-based adolescents in Japan. *Methods*. Study subjects comprised adolescents in the seventh grade (age range, 12–13 years) from Ina, a town in Saitama Prefecture, Japan, between 1999 and 2008. The height and weight of the subjects were measured, and information concerning eating behaviors (eating speed and eating until full) was obtained using a self-administered questionnaire. *Results*. Among boys (*n* = 1586), fast eating speed significantly increased the odds ratio (OR) for overweight when compared with medium eating speed, regardless of eating until full or not; moreover, a more marked increase in the OR was observed among boys eating until full (OR: 2.78, 95% confidence interval: 1.76–4.38) than among those not eating until full (2.43, 1.41–4.20). Among girls (*n* = 1542), fast eating speed led to a significant increase in the OR in those eating until full; however, no significant increases were observed in the OR in those eating quickly and not until full. *Conclusions*. Among adolescents, fast eating speed was associated with overweight; furthermore, the combination of both fast eating speed and eating until full may have a significant effect on overweight.

## 1. Introduction

Obesity is a major risk factor for chronic diseases, and it also plays a central role in both insulin resistance and metabolic syndrome, which includes hyperinsulinemia, hypertension, hyperlipidemia, and type 2 diabetes mellitus [1]. It was recently reported that the metabolic and physiologic abnormalities associated with obesity in adolescence (e.g., hypertension, dyslipidemia, and type 2 diabetes) tend to track into adulthood, along with the condition of obesity itself [2]. Moreover, independent of adult obesity status, adolescent obesity increases the long-term risk of adult mortality and morbidity [3]. Therefore, the importance of obesity prevention in adolescents is evident.

Overweight and obesity have also been reported to be associated with a variety of lifestyle factors [4–13], among which, eating behavior has long been identified as a factor. Several studies have reported an association between eating speed and overweight or obesity [9–11], and eating until full, which refers to consuming a large quantity of food in one meal and is unrelated to eating disorders [12], has been reported to be associated with overweight [12, 13]. However, the effect of eating speed on overweight could depend on the behavior of eating until full, because the total energy intake in those who eat quickly and until full is usually higher than that in those who eat quickly but not until full. In fact, Maruyama et al. reported that both eating quickly and eating until full are associated with overweight among adults, and the combination of the two may have a substantial impact on overweight [12]. For example, fast eating speed may not have a significant effect on overweight if the individual does not eat until full. Therefore, when eating speed is evaluated as a risk factor for overweight or obesity, it may be essential to consider eating until full behavior. However, to the best of our knowledge, the combined effect of eating speed and eating until full behavior on overweight among adolescents has yet to be reported.

Accordingly, the aim of the present study was both to investigate the relationship between eating behaviors (eating speed or eating until full) and overweight and to examine the effect of the combined behaviors on overweight among population-based adolescents in Japan.

## 2. Methods

In addition to the annual national health checkups performed in accordance with the School Health Law of Japan, the town of Ina, located in Saitama Prefecture, has conducted a unique health-promotion program since 1994.

The program consists of a questionnaire survey, blood test, and physical examination for both fourth and seventh graders. Several studies concerning this program have been conducted [14–16].

### 2.1. Study Subjects

Study subjects comprised a total of 3256 seventh-grade school children (age range, 12-13 years) from Ina between 1999 and 2008 (*n* = 306 in 1999; 309 in 2000; 298 in 2001; 317 in 2002; 293 in 2003; 315 in 2004; 312 in 2005; 354 in 2006; 380 in 2007; and 372 in 2008). Informed consent was obtained from the parent or guardian of each subject prior to their participation in the study. The study protocol was approved by the Medical Ethics Committee of Showa University School of Medicine.

### 2.2. Questionnaire Survey

A self-administered questionnaire was distributed to each subject by a teacher in the junior high school. Each subject and a parent or guardian completed the questionnaire, which was composed of the following two sections: one (on the front of the questionnaire) to be completed by the subject; and the other (on the back) to be completed by the parent or guardian.

The questionnaire asked subjects about the following items: sex; age; exercise other than physical education class (daily, sometimes, or none); snacking after dinner (always, often, seldom, or none); eating speed; and eating until full behavior. Information concerning eating speed was based on three possible responses (fast, medium, or slow) [17] to the question “How fast is your eating speed compared to others?” With regard to eating until full behavior, possible responses were either yes or no [18].

The parent or guardian of each subject was asked to complete a self-administered questionnaire regarding the subject's birth weight, wake-up time, bedtime, frequency of eating breakfast (daily, sometimes, or none), and whether the child was an only child. The heights and weights of the parents or guardians were also self-reported in the questionnaire. Self-reported height and weight are commonly used in epidemiological studies and are generally reliable in Japanese men and women [19]. Sleep duration was calculated from wake-up time and bedtime, and frequency of eating breakfast was categorized into the following two groups: skipping breakfast (sometimes and none) and not skipping breakfast (daily).

### 2.3. Anthropometric Measurements

To protect the privacy of the subjects, height and weight were measured either in the school's infirmary or in a designated room. For the anthropometric measurements, the subjects wore light clothing and were barefoot. Height was measured to the nearest 0.1 cm using a stadiometer, and body weight was measured to the nearest 0.1 kg using a scale. Body mass index (BMI) was calculated as weight (kg) divided by height (m) squared. These measurements were recorded annually from 1999 to 2008.

### 2.4. Definition of Overweight and Obesity

Childhood overweight (including obesity) was determined according to the age- and sex-specific cut-off points proposed by the International Obesity Task Force [20]. The criteria have been used in numerous epidemiological studies for both Japanese children and adolescents [8, 21–24]. Based on previous studies for Japanese adults [25, 26], parents who had a BMI ≥25 kg/m^2^ were defined as obese.

### 2.5. Data Analysis

Statistical analysis was performed separately for each sex. Either the chi-square test or the Wilcoxon rank-sum test was used to compare various characteristics between the overweight and nonoverweight groups. A logistic regression model was then employed to evaluate the relationship between eating behaviors and overweight, and both the crude odds ratio (OR) for overweight and the 95% confidence interval (95% CI) were estimated and subsequently adjusted for potential confounders. Variables that had been reported to be associated with overweight [4, 8, 27–30], and that were different between the overweight and the nonoverweight groups (*P* value less than 0.05) in this study, were considered as potential confounders. These included birth weight, obesity status of the parent, frequency of exercise, and skipping breakfast. A *P* value less than 0.05 was considered statistically significant. All statistical analyses were performed using Statistical Analysis System (SAS) software (version 9.2; SAS Institute Inc., Cary, NC, USA).

## 3. Results

Among all 3256 subjects, 28 refused to participate in the program (participation rate: 99.1%), and 100 were excluded due to incomplete data. Thus, data from a total of 3128 subjects were analyzed.

The characteristics of both the overweight and the nonoverweight boys (*n* = 1586) are shown in Table 1. Boys with one or more obese parents were more frequently found to be in the overweight group, and statistically significant differences were observed between the overweight and nonoverweight groups in both birth weight and frequency of exercise. A significantly higher proportion of those who eat quickly was found in the overweight group when compared with the nonoverweight group. Furthermore, a higher proportion of subjects who reported eating until full was found in the overweight group; however, this difference was not statistically significant.

The characteristics of both the overweight and nonoverweight girls (*n* = 1542) are shown in Table 2. Similar to boys, girls with one or more obese parents were more frequently found to be in the overweight group, and statistically significant differences were also observed between the overweight and nonoverweight groups in both birth weight and frequency of exercise. A higher proportion of those who skip breakfast and a higher proportion of those who eat quickly were found in the overweight group.

The crude and adjusted ORs of eating speed or eating until full for overweight are shown in Table 3. When compared to adolescents who reported a medium eating speed, a significantly increased OR was found among both boys (OR: 2.65, 95% CI: 1.87–3.75, *P* < 0.001) and girls (1.73, 1.05–2.85, *P* = 0.033) in the fast eating speed group, whereas no increased OR was observed in the slow eating group, regardless of sex. The adjusted OR of eating until full was not statistically significant for either sex.

The adjusted ORs of eating speed for overweight were then calculated based on eating until full behavior (Table 4). In boys, a significant increase in the OR of a fast eating speed was observed compared with a medium eating speed, regardless of eating until full behavior. Furthermore, a more marked increase was observed among those eating until full (OR: 2.78, 95% CI: 1.76–4.38, *P* < 0.001) than among those not eating until full (2.43, 1.41–4.20, *P* = 0.001) in the OR for overweight. In girls, fast eating speed resulted in a significantly increased OR among those eating until full, whereas no significant increase was observed in OR among those not eating until full. The ORs of eating slowly did not increase among either sex, regardless of eating until full behavior.

## 4. Discussion

In this study, some significantly different baseline characteristics were observed between the overweight and nonoverweight groups. For example, adolescents with one or more obese parents were more frequently found in the overweight group. Familial variables, especially parental obesity, have been reported to be the most important in relation to childhood obesity [31], and the association between parental overweight/obesity and childhood overweight/obesity has been reported in a number of studies [7, 8, 27, 30]. Moreover, a relationship has also been reported between overweight and birthweight, exercise, or skipping breakfast among children or adolescents [4, 8, 29, 30]. Therefore, our results were consistent with those from previous studies.

Furthermore, as shown in Table 3, fast eating speed significantly increased the OR for overweight, which is also consistent with results from previous studies [8, 11, 12]. Fast eating speed has been shown to be associated with adolescent overweight [8] and associated with overweight in children [11]. It has also been shown to be a risk factor for obesity among preschool children [10]. One possible mechanism underlying the relationship between fast eating speed and overweight is the increase in energy intake among fast eaters; this is because energy intake per day increases significantly with an increase in eating speed [9]. In addition, sex differences were observed in the effect of fast eating speed on overweight (Table 3). The adjusted OR of fast eating speed for overweight among boys was higher than that observed among girls. One of the reasons could be the difference between the sexes in eating speed. A previous study reported that men ate faster than women and women took more bites and longer to eat than men [32]. The second reason might be the result of differences between the sexes in food preferences. Boys have been shown to prefer meat, processed meat products, eggs, and fatty and sugary foods more than girls [33]. In addition, girls may be more likely to select low calorie foods when they snack due to girls' preferences in relation to body shape [8]. Accordingly, the differences observed between the sexes in the effect of eating speed on overweight may be the result of the differences in eating speed and/or food preferences; however, further study is needed to elucidate the biological mechanisms underlying these differences.

In this study, the effect of eating quickly on overweight varied by eating until full behavior (Table 4). The adjusted OR of fast eating speed for overweight was higher among those eating until full than that among those not eating until full. Eating quickly can lead to a reduced awareness of the quantity of food consumed, which in turn can lead to consumption of a quantity of food that exceeds the amount necessary for satiety [34]. It has recently been reported that fast eating speed may lead to overeating before the stomach senses fullness [9, 11]. Moreover, total energy intake has been shown to be higher among individuals who report eating until full than among those who do not [12]. Therefore, the amount of food consumed by those who eat quickly and until full could be larger than that consumed by those who eat quickly but do not eat until full. In girls, eating both quickly and until full was associated with an increase in the OR for overweight, whereas eating quickly and not until full did not. These results suggest that the combination of eating quickly and until full has a substantial effect on overweight, while not eating until full helps to prevent overweight in girls. However, further study is needed to verify these results because information on total energy intake was not collected in this study.

On the other hand, regardless of eating until full or not, no increase was observed in the OR of slow eating speed for overweight. Eating slowly may help to maximize satiation and thus reduce energy intake during meals [35]. In fact, it was recently reported that linear eaters—that is, people who eat at an approximately constant rate—ate less food when challenged to eat at a lower speed [36]. Furthermore, it has been shown that, by eating more slowly, an obese patient can improve his digestion, learn to savor the food, and may eventually achieve a normal state of satiation with less food intake [37]. Therefore, eating slowly could be effective for the prevention of overeating and childhood overweight.

The strengths of this study were that the outcome (overweight or nonoverweight) was defined by height and weight measurements, and that the effect of the combinations of eating speed and eating until full on overweight was evaluated in over 3000 population-based adolescents. However, this study has some potential limitations. First, information concerning eating speed and eating until full was self-reported and, thus, could be not objectively evaluated. However, a high level of concordance between self-reported and friend-reported rates of eating has been reported [38]. Additionally, a statistically positive association between the self-reported rate of eating and energy intake has been recently reported [9], and self-reported rapid eating has been shown to be associated with both overweight and weight gain [8, 11, 34]. Therefore, the findings from this study could be considered reasonable; however, further prospective or intervention studies are needed to verify our results. Second, the possibility of residual confounding in this study cannot be excluded. For instance, socioeconomic status (SES), which has been reported as being a risk factor for both overweight and obesity [39, 40], was not included as an item in the study questionnaire and therefore not evaluated. Third, the present study results were based on data from only one town in Japan. Therefore, due to cultural differences in eating behavior, applying our results to other populations may be difficult. Finally, the present study was a cross-sectional study, and, thus, determining a causal relationship of eating behaviors to overweight was not possible. Therefore, the possibility of reverse causality cannot be excluded.

## 5. Conclusion

Results of the present study indicated that fast eating speed was associated with overweight. Moreover, the combination of both fast eating speed and eating until full may have a significant effect on overweight among adolescents. Decreasing eating speed is therefore suggested to be an effective strategy for the prevention of overweight among adolescents.

## Figures and Tables

**Table 1:** Characteristics of the nonoverweight and the overweight boys among study participants.

Variables	Nonoverweight (*n* = 1355)	Overweight (*n* = 231)	*P* value^a^
Age (years)	12.0 (12.37)	12.0 (12.39)	0.594
Birthweight (g)			
<2500	6.0	8.9	<0.001
2500–2999	29.1	23.0	
3000–3499	45.7	38.1	
3500–3999	17.4	24.8	
4000+	1.8	5.3	
Only child (%)	7.8	9.3	0.438
Parent's obesity (%)			
None	69.5	41.2	<0.001
Father only	20.9	32.3	
Mother only	5.6	16.2	
Father and mother	4.1	10.4	
Exercise (%)			
None	9.5	16.1	0.005
Sometimes	8.4	10.3	
Daily	82.1	73.5	
Sleeping hours (%)			
9.0+	24.4	21.1	0.785
8.0–8.9	53.5	55.4	
7.0–7.9	19.8	21.1	
<7.0	2.3	2.5	
Skipping breakfast (%)	5.2	4.4	0.624
Snack after dinner (%)			
Seldom or none	39.9	42.6	0.441
Always or often	60.1	57.4	
Eating speed (%)			
Fast	21.8	47.2	<0.001
Medium	59.6	46.3	
Slow	18.6	6.5	
Eating until full (%)	54.7	58.9	0.237

Except where indicated percentage (%), values are median (mean).

^a^Chi-square test or Wilcoxon rank-sum test.

**Table 2:** Characteristics of the nonoverweight and the overweight girls among study participants.

Variables	Nonoverweight (*n* = 1363)	Overweight (*n* = 179)	*P* value^a^
Age (years)	12.0 (12.36)	12.0 (12.37)	0.757
Birthweight (g)			
<2500	7.3	1.7	<0.001
2500–2999	35.7	29.3	
3000–3499	44.0	47.1	
3500–3999	11.9	16.1	
4000+	1.1	5.8	
Only child (%)	8.2	11.2	0.175
Parent's obesity (%)			
None	67.4	41.8	<0.001
Father only	21.9	25.5	
Mother only	8.2	23.4	
Father and mother	2.5	9.2	
Exercise (%)			
None	26.4	38.5	<0.001
Sometimes	13.5	20.7	
Daily	60.1	40.8	
Sleeping hours (%)			
9.0+	15.5	12.5	0.610
8.0–8.9	50.7	50.7	
7.0–7.9	29.5	33.6	
<7.0	4.2	3.3	
Skipping breakfast (%)	7.2	11.8	0.033
Snack after dinner (%)			
Seldom or none	43.0	41.5	0.700
Always or often	57.0	58.5	
Eating speed (%)			
Fast	11.7	18.4	0.010
Medium	62.4	63.1	
Slow	25.3	18.4	
Eating until full (%)	53.3	50.8	0.541

Except where indicated percentage (%), values are median (mean).

^a^Chi-square test or Wilcoxon rank-sum test.

**Table 3:** Crude and adjusted odds ratios of eating speed or eating until full for being overweight.

Variables	Total *N*	Overweight *n* (%)	Crude		Adjusted	
			OR (95% CI)	*P* value	OR (95% CI)	*P* value
Among boys						
Eating speed						
Fast	404	109 (27.0)	2.79 (2.07–3.76)	<0.001	2.65 (1.87–3.75)	<0.001
Medium	915	107 (11.7)	1.00		1.00	
Slow	267	15 (5.6)	0.45 (0.26–0.79)	0.005	0.51 (0.27–0.94)	0.031
Eating until full						
Yes	877	136 (15.5)	1.19 (0.89–1.57)	0.237	1.15 (0.83–1.60)	0.394
No	709	95 (13.4)	1.00		1.00	
Among girls						
Eating speed						
Fast	193	33 (17.1)	1.55 (1.02–2.37)	0.041	1.73 (1.05–2.85)	0.033
Medium	964	113 (11.7)	1.00		1.00	
Slow	385	33 (8.6)	0.71 (0.47–1.06)	0.094	0.74 (0.45–1.20)	0.218
Eating until full						
Yes	817	91 (11.1)	0.91 (0.66–1.24)	0.541	0.84 (0.58–1.22)	0.353
No	725	88 (12.1)	1.00		1.00	

OR: odds ratio; CI: confidence interval. Adjusted for birth weight, parents' obesity, exercise, and skipping breakfast.

**Table 4:** Adjusted odds ratios of eating speed for being overweight by eating until full or not.

Variables	Eating until full				Not eating until full			
	Total *N*	Overweight *n* (%)	AOR (95% CI)	*P* value	Total *N*	Overweight *n* (%)	AOR (95% CI)	*P* value
Among boys								
Eating speed								
Fast	242	71 (29.3)	2.78 (1.76–4.38)	<0.001	162	38 (23.5)	2.43 (1.41–4.20)	0.001
Medium	503	56 (11.1)	1.00		412	51 (12.4)	1.00	
Slow	132	9 (6.8)	0.57 (0.25–1.28)	0.170	135	6 (4.4)	0.42 (0.16–1.11)	0.080
Among girls								
Eating speed								
Fast	106	22 (20.8)	2.97 (1.53–5.76)	0.001	87	11 (12.6)	0.90 (0.39–2.05)	0.793
Medium	503	50 (9.9)	1.00		461	63 (13.7)	1.00	
Slow	208	19 (9.1)	0.87 (0.43–1.75)	0.689	177	14 (7.9)	0.65 (0.33–1.30)	0.227

AOR: adjusted odds ratio; CI: confidence interval. Adjusted for birth weight, parents' obesity, exercise, and skipping breakfast.

## References

[1] Kelishadi R (2007). Childhood overweight, obesity, and the metabolic syndrome in developing countries. *Epidemiologic Reviews*.

[2] Story M, Sallis JF, Orleans CT (2009). Adolescent obesity: towards evidence-basedpolicy and environmental solutions. *Journal of Adolescent Health*.

[3] Must A, Jacques PF, Dallal GE, Bajema CJ, Dietz WH (1992). Long-term morbidity and mortality of overweight adolescents—a follow-up of the Harvard Growth Study of 1922 to 1935. *The New England Journal of Medicine*.

[4] Janssen I, Katzmarzyk PT, Boyce WF, King MA, Pickett W (2004). Overweight and obesity in Canadian adolescents and their associations with dietary habits and physical activity patterns. *Journal of Adolescent Health*.

[5] Patrick K, Norman GJ, Calfas KJ (2004). Diet, physical activity, and sedentary behaviors as risk factors for overweight in adolescence. *Archives of Pediatrics and Adolescent Medicine*.

[6] Berkey CS, Rockett HR, Field AE (2000). Activity, dietary intake, and weight changes in a longitudinal study of preadolescent and adolescent boys and girls. *Pediatrics*.

[7] Agras WS, Hammer LD, McNicholas F, Kraemer HC (2004). Risk factors for childhood overweight: a prospective study from birth to 9.5 years. *Journal of Pediatrics*.

[8] Sun Y, Sekine M, Kagamimori S (2009). Lifestyle and overweight among Japanese adolescents: the Toyama Birth Cohort Study. *Journal of Epidemiology*.

[9] Otsuka R, Tamakoshi K, Yatsuya H (2006). Eating fast leads to obesity: findings based on self-administered questionnaires among middle-aged Japanese men and women. *Journal of Epidemiology*.

[10] He Q, Ding ZY, Fong DYT, Karlberg J (2000). Risk factors of obesity in preschool children in China: a population-based case—control study. *International Journal of Obesity*.

[11] Sugimori H, Yoshida K, Izuno T (2004). Analysis of factors that influence body mass index from ages 3 to 6 years: a study based on the Toyama cohort study. *Pediatrics International*.

[12] Maruyama K, Sato S, Ohira T (2008). The joint impact on being overweight of self reported behaviours of eating quickly and eating until full: cross sectional survey. *British Medical Journal*.

[13] Kimura Y, Nanri A, Matsushita Y, Sasaki S, Mizoue T (2011). Eating behavior in relation to prevalence of overweight among Japanese men. *Asia Pacific Journal of Clinical Nutrition*.

[14] Ochiai H, Shirasawa T, Nishimura R (2010). Relationship of body mass index to percent body fat and waist circumference among schoolchildren in Japan—the influence of gender and obesity: a population-based cross-sectional study. *BMC Public Health*.

[15] Shirasawa T, Shimada N, Ochiai H (2010). High blood pressure in obese and nonobese Japanese children: blood pressure measurement is necessary even in nonobese Japanese children. *Journal of Epidemiology*.

[16] Nishimura R, Sano H, Matsudaira T (2009). Changes in body mass index, leptin and adiponectin in Japanese children during a three-year follow-up period: a population-based cohort study. *Cardiovascular Diabetology*.

[17] Tanihara S, Imatoh T, Miyazaki M (2011). Retrospective longitudinal study on the relationship between 8-year weight change and current eating speed. *Appetite*.

[18] Tomofuji T, Furuta M, Ekuni D (2011). Relationships between eating habits and periodontal condition in university students. *Journal of Periodontology*.

[19] Wada K, Tamakoshi K, Tsunekawa T (2005). Validity of self-reported height and weight in a Japanese workplace population. *International Journal of Obesity*.

[20] Cole TJ, Bellizzi MC, Flegal KM, Dietz WH (2000). Establishing a standard definition for child overweight and obesity worldwide: international survey. *British Medical Journal*.

[21] Murakami K, Miyake Y, Sasaki S, Tanaka K, Arakawa M (2012). Self-reported rate of eating and risk of overweight in Japanese children: Ryukyus Child Health Study. *Journal of Nutritional Science and Vitaminology*.

[22] Kouda K, Fujita Y, Nakamura H, Takeuchi H, Iki M (2011). Effect of recovery from obesity on cardiovascular risk factors among Japanese schoolchildren: the Iwata population-based follow-up study. *Journal of Epidemiology*.

[23] Sekine M, Yamagami T, Handa K (2002). A dose-response relationship between short sleeping hours and childhood obesity: results of the Toyama Birth Cohort Study. *Child*.

[24] Sekine M, Yamagami T, Hamanishi S (2002). Parental obesity, lifestyle factors and obesity in preschool children: results of the Toyama Birth Cohort Study. *Journal of Epidemiology*.

[25] Examination Committee of Criteria for “Obesity Disease” in Japan (2002). New criteria for “obesity disease” in Japan. *Circulation Journal*.

[26] Matsuzawa Y, Inoue S, Ikeda Y (2000). New diagnostic criteria of obesity. *Journal of Japan Society for the Study of Obesity*.

[27] Thibault H, Contrand B, Saubusse E, Baine M, Maurice-Tison S (2010). Risk factors for overweight and obesity in French adolescents: physical activity, sedentary behavior and parental characteristics. *Nutrition*.

[28] Júlíusson PB, Eide GE, Roelants M, Waaler PE, Hauspie R, Bjerknes R (2010). Overweight and obesity in Norwegian children: prevalence and socio-demographic risk factors. *Acta Paediatrica*.

[29] Apfelbacher CJ, Loerbroks A, Cairns J, Behrendt H, Ring J, Krämer U (2008). Predictors of overweight and obesity in five to seven-year-old children in Germany: results from cross-sectional studies. *BMC Public Health*.

[30] Parsons TJ, Power C, Logan S, Summerbell CD (1999). Childhood predictors of adult obesity: a systematic review. *International Journal of Obesity*.

[31] Dietz W (1991). Factors associated with childhood obesity. *Nutrition*.

[32] Hill SW, McCutcheon NB (1984). Contributions of obesity, gender, hunger, food preference, and body size to bite size, bite speed, and rate of eating. *Appetite*.

[33] Cooke LJ, Wardle J (2005). Age and gender differences in children’s food preferences. *British Journal of Nutrition*.

[34] Gerace TA, George VA (1996). Predictors of weight increases over 7 years in fire fighters and paramedics. *Preventive Medicine*.

[35] Andrade AM, Greene GW, Melanson KJ (2008). Eating slowly led to decreases in energy intake within meals in healthy women. *Journal of the American Dietetic Association*.

[36] Ioakimidis I, Zandian M, Bergh C, Södersten P (2009). A method for the control of eating rate: a potential intervention in eating disorders. *Behavior Research Methods*.

[37] Stuart RB (1967). Behavioral control of overeating. *Behaviour Research and Therapy*.

[38] Sasaki S, Katagiri A, Tsuji T, Shimoda T, Amano K (2003). Self-reported rate of eating correlates with body mass index in 18-y-old Japanese women. *International Journal of Obesity*.

[39] Kleiser C, Schaffrath Rosario A, Mensink GB, Prinz-Langenohl R, Kurth BM (2009). Potential determinants of obesity among children and adolescents in Germany: results from the cross-sectional KiGGS Study. *BMC Public Health*.

[40] Danielzik S, Czerwinski-Mast M, Langnäse K, Dilba B, Müller MJ (2004). Parental overweight, socioeconomic status and high birth weight are the major determinants of overweight and obesity in 5–7 y-old children: baseline data of the Kiel Obesity Prevention Study (KOPS). *International Journal of Obesity*.

